# Supplementary material for: High-frequency recombination between members of an LTR retrotransposon family during transposition bursts
Source: Nat Commun. 2017 Nov 3;8:1283. doi: 10.1038/s41467-017-01374-x (PMC5668417; doi:10.1038/s41467-017-01374-x)
Supplement: Supplementary file 1 — Supplementary Information [file 41467_2017_1374_MOESM1_ESM.pdf]

Supplementary Fig. 1

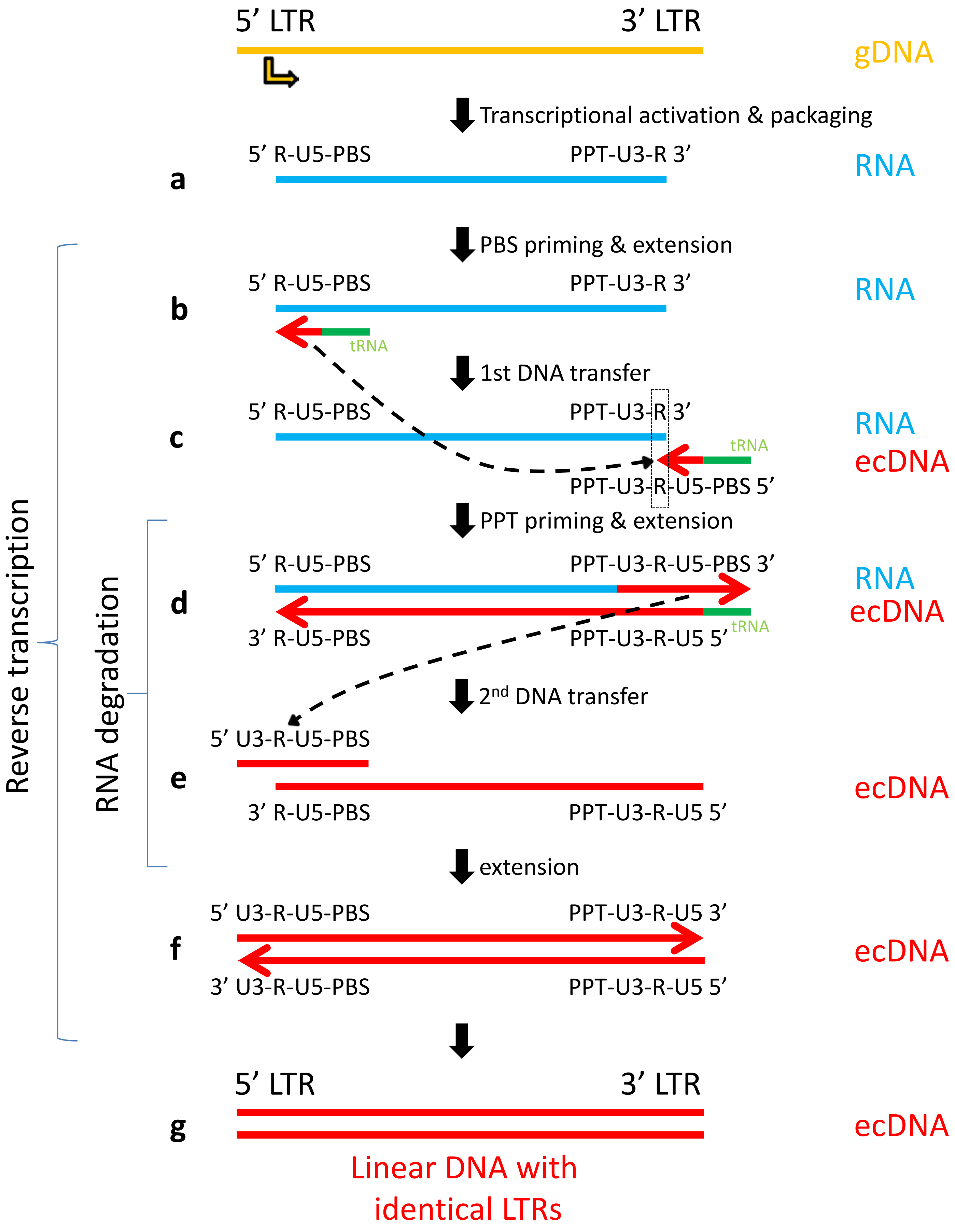

Supplementary Fig. 1

Supplementary Fig. 1: Schematic modelled representation of reverse transcription steps of LTR retrotransposons. LTR - long terminal repeat. R - repeated region, U5 - unique 5’ region, U3 - unique 3’ region, PBS - primer binding site, PPT - polypurine track, ecDNA - extra-chromosomal DNA. (**a**) The LTR retrotransposon is transcriptionally activated and the RNA genome (starting and ending in the LTR R region) is packaged with reverse transcriptase, RNAseH and integrase into GAG-derived virus-like particles. (**b**) Host tRNA primed at PBS and (-)-strand cDNA is synthesized by reverse transcriptase. (**c**) (-)-strand cDNA-tRNA transfer using sequence homologies in the R region. (**d**) Reverse transcriptase extends (-)-strand cDNA until the beginning of the transcript, after RNAseH degradation of the RNA, except at the PPT, where synthesis of (+)-strand cDNA is primed and synthetized using (-)-strand cDNA-tRNA as a template. (**e**) (+)-strand cDNA transfers to 5’ area of (-)-strand cDNA. (**f**) Reverse transcriptase performs final extensions. (**g**) The resulting ecDNA molecule is a double-stranded, linear molecule encoding the entire retrotransposon with two identical LTRs.

Supplementary Fig. 2

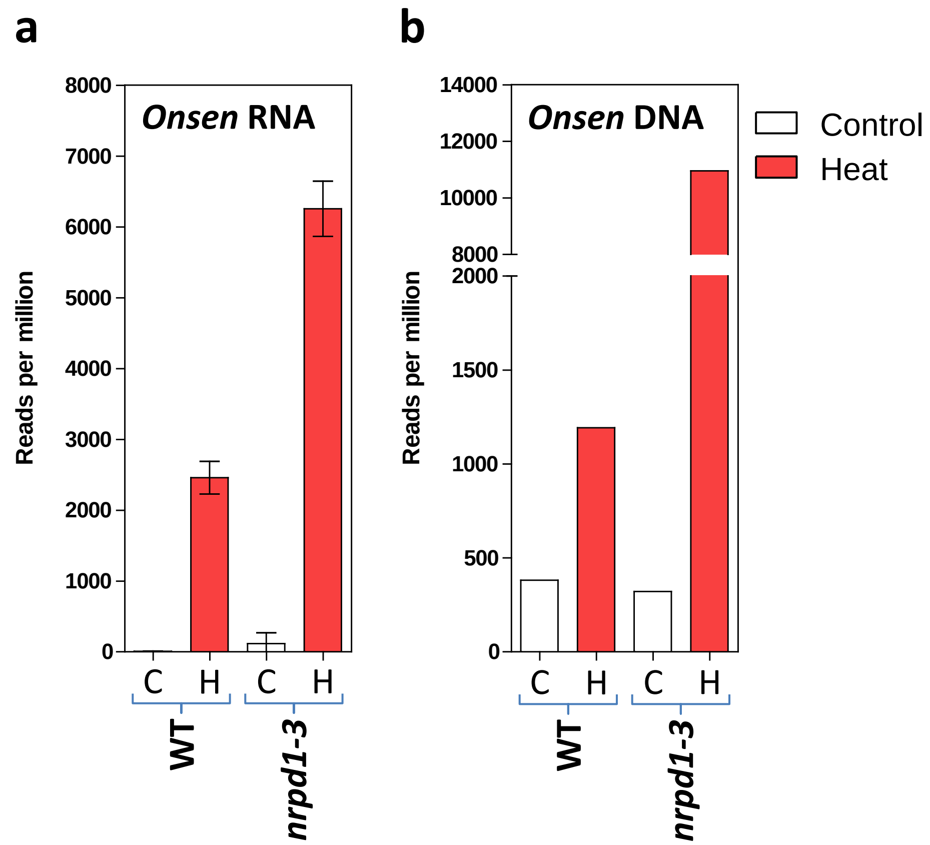

Supplementary Fig. 2: Heat stress induced increase of reads mapping to *Onsen* in the NGS transcriptome and whole-genome sequencing. Total NGS reads mapping to *Onsen* were counted and normalized to library size in wild-type and *nrpd1-3* plants under control (white bars) and heat stress (red bars) conditions. (**a**) RNA-seq analysis. The increased reads in heat samples originated from transcriptionally activated *Onsen*. Data represent means ± SEM for n=2 independent biological replicates. (**b**) Whole-genome sequencing analysis. The increased reads in heat samples originated from activated *Onsen* producing ecDNA.

Supplementary Fig. 3

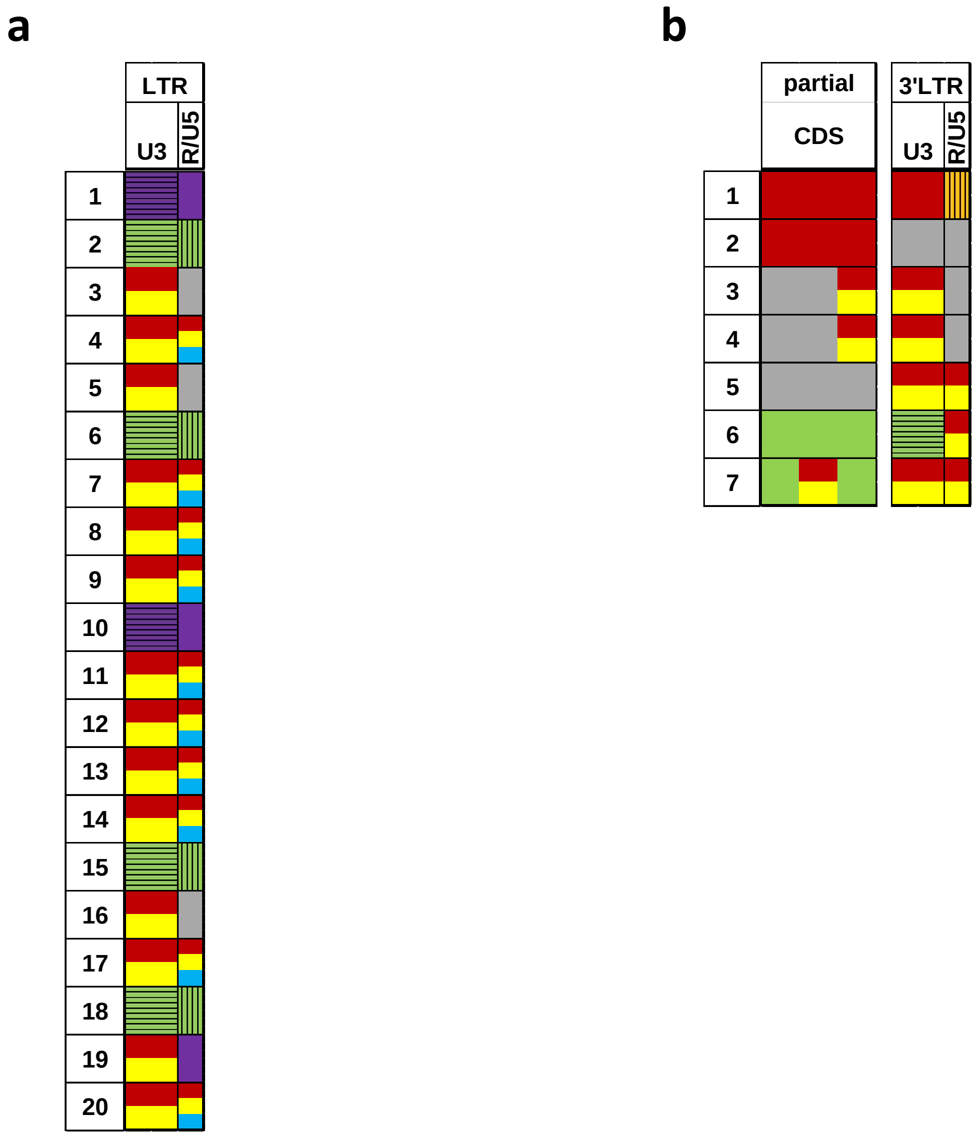

Supplementary Fig. 3: Independent plant lines showing evidence of *Onsen* intra-family recombination. (**a**) Origin of reconstructed LTRs from new chromosomal *Onsen* insertions recovered in progeny of an independent plant (*nrpd1* plant 5L2) with 20 new insertions [8]. LTRs domains are marked as U3 and R/U5. Informative SNPs and indels were used to infer the parental origin of LTRs. Several colours are used in the same area when these polymorphisms are shared between more than one member. Colour code as in Fig. 1a. (**b**) First cloned and Sanger-sequenced new chromosomal *Onsen* insertions from progeny of independent heat stressed *nrpd1-3* mutant plants (initial experiments). Informative SNPs and indels were used to determine the parental origin of new inserted retrotransposon copies. The sequenced area was between positions 3701 and 4957, when aligned to AT1G11265/'red'. Retrotransposon domains and colour code as in Fig. 1a.

Supplementary Fig. 4

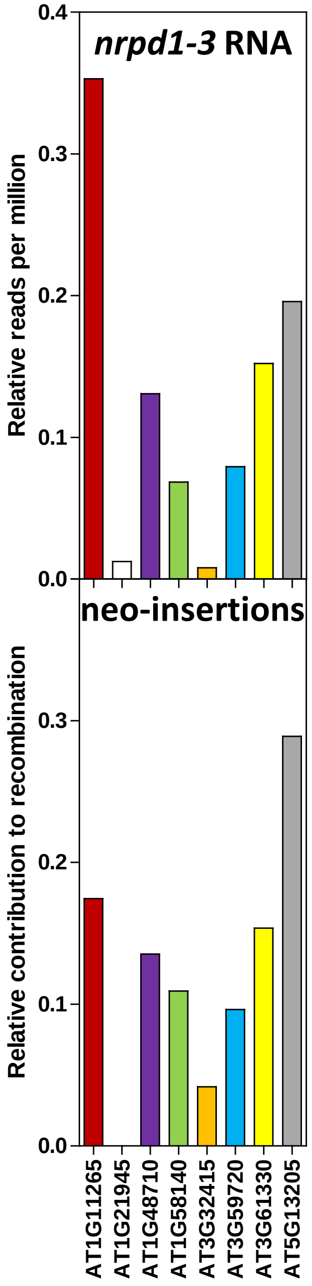

<u>Supplementary Fig. 4</u>: Relative contributions of family members to the *Onsen* mRNA pool and to the 32 cloned and Sanger-sequenced new insertions. Mean relative transcript levels (upper panel) were calculated from Fig. 1b; the relative contribution to neo-insertions (lower panel) was computed with a sum of scores as described in Methods. Colour code as in Fig. 1a.

Supplementary Fig. 5

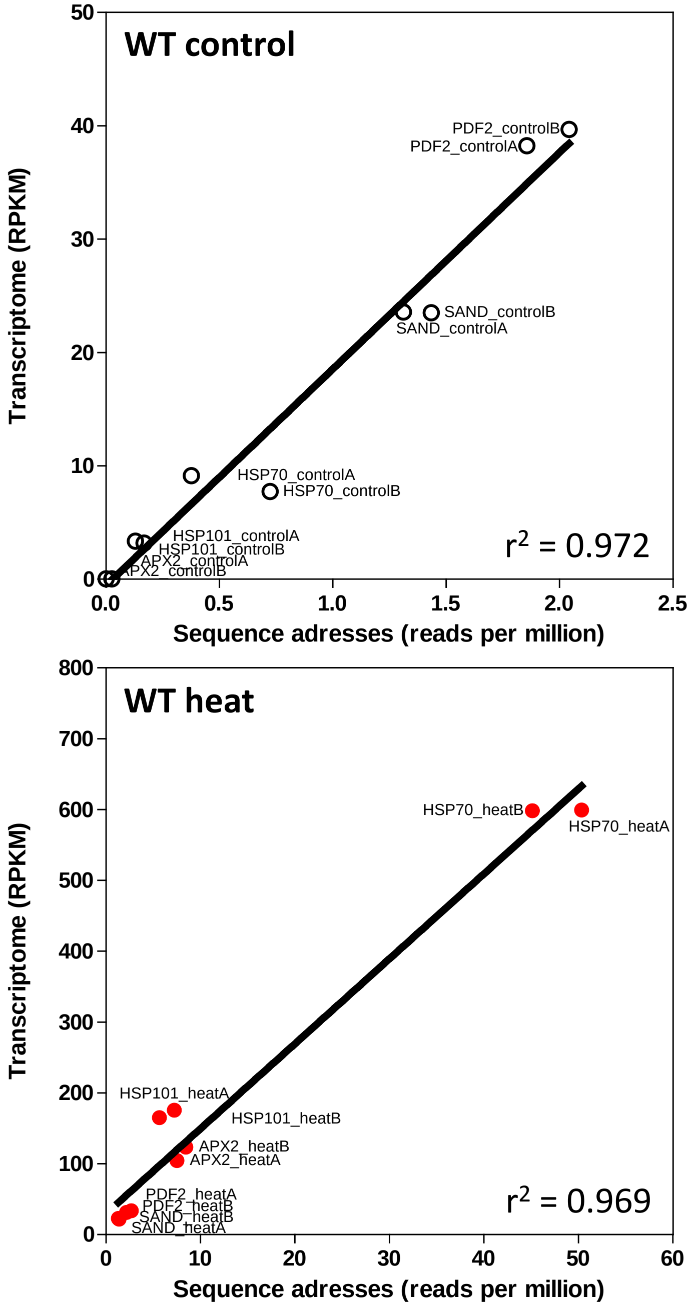

Supplementary Fig. 5: Validation of the sequence addresses strategy to estimate transcript levels. In both control (upper panel) and heat stress (lower panel) samples, standard determination of transcript levels (RPKM) was calculated from transcriptome data of wild-type samples and correlated with the normalized read count of addresses across case examples of housekeeping and heat responsive genes.

Supplementary Table 1

Non-parentally originated polymorphism events observed in cloned and Sanger-sequenced *Onsen* new chromosomal copies. ‘Position’ refers to alignment on AT1G11265/’red’ as a reference.

**New copy *Onsen***	**Event**	**Position (alignment)**
#2	G to T	3729 - ORF
#8	Deletion+SNPs	456 to 503 - ORF
#13	G to A	3598 - ORF
#17	T to C	148 and 4664 - LTR
#18	T to C	3121 - ORF
#18	Insertion A	4910 - ORF
#26	T to C	3322 - ORF
#26	C to T	4211 - ORF
#30	A to T	1598 - ORF
#30	G to A	1874 - ORF

Supplementary Table 2
List of primers used.

For fig. 3, cloning and Sanger-sequencing of *Onsen* new insertions in *nrpd1* 2L plant

	Insertion - Position	Primer Forward	Sequence (5'-3')	Primer Reverse	Sequence (5'-3')
1	exon AT1G17430	NI_1F	GTGGAGTGGGATTTACACAGCAGCA	NI_1R	AACGAACAAACAATTTACGGGACCAGA
2	exon AT1G58050	NI_2F	CTAAAGGCACCCGCAGCATCTC	NI_2R	TTGTCAAGCATGGTGGAAGATTGG
3	exon AT3G47760	NI_3F	TCCTTCTCCATATGGCTTGCAAAGA	NI_3R	TCACATTGGAAGTGAGATGAGCCCTA
4	exon AT4G29110	NI_4F	ATAACCAACCTCCGCCGTCGAT	NI_4R	CGAATCCGTCACCCTGAGCTGT
5	exon AT1G61470	NI_5F	CCAGCAGCTTTTGGGAAGGTTTG	NI_5R	TTGCTGATGCTGCTGCTGCTCT
6	exon AT1G12140	NI_6F	ACCGAAGTAGTTCTTGTCGAGCCTGA	NI_6R	TGTTTCCATCGTCAAAAACGCACA
7	3'UTR AT2G22330	NI_7F	GGTGAAGTGAGGCGACGGTGAC	NI_7R	TTGTCGCGACCCAATCATTGAC
8	exon AT4G34530	NI_8F	TGGCTTATCTGTTTCTTCTGTGTGTGC	NI_8R	GCCTGTGATCTTGTCGCATCCA
9	exon AT4G31690	NI_9F	ACCACTCTCTTCGCCTTCTTCATGTC	NI_9R	GAGCACATCGAGGGAAAACACGA
10	exon AT2G36570	NI_10F	CCTTCCCTCTCCTTACGAGGACCA	NI_10R	CCGGATCGTTAAAACCCGGGTAAA
11	exon AT1G11370	NI_11F	TCAAACAGTTGAATGAGTATGCAAAAGATGA	NI_11R	ACCACGGTGAATGAAGCGGTTG
12	exon AT2G14285	NI_12F	GCGCAGACCCTCAAGCATCATC	NI_12R	TGAATGAAGCTTGGTTTGTAATTGAACGTC
13	exon AT3G23060	NI_13F	CGTTCCAGAAACGTGGGAGAAAA	NI_13R	TGGTACGACTTCCTGAAAAGTTGATCC
14	exon AT5G39310	NI_14F	TCCACCATTGATGTCACCGTAGAATG	NI_14R	TCCCAAAAATATCGACAAGCCCAAA
15	exon AT3G04010	NI_15F	TTTCAGATCACTACCCAATATCTAAAAAC	NI_15R	TGAATTATCGACGAAAACAGAGTGC
16	5'UTR AT4G35770	NI_16F	GTCCGAGTCAGCAAGTTATCCTCTT	NI_16R	TTCTCGTCGTTGATTGCTAGCTTAC
17	exon AT5G22250	NI_17F	CCCATCCTCAACGAAATACAAATC	NI_17R	GGATCGATTTCGAGCGGAAC
18	exon AT5G13940	NI_18F	TCTCATTCAAACAAAAACTAAAAGTGGAA	NI_18R	TGCAATAACCGGCTTGTTGACTC
19	exon AT2G16960	NI_19F	GGGGTTCAATACGACCAAAATATCG	NI_19R	TCCGGGAGTACCTGTTAGTGTAGCC
20	intron AT2G35640	NI_20F	TTAGGATTTGTAGCTCCGATGATGG	NI_20R	CGACCAAAATGCGGGTTTAATTACT
21	upstream AT1G51970	NI_21F	GTTTCCCAAATTCTTGACCAAACC	NI_21R	TGTGTACATATAAACATAAACGGTAAGTCG
22	exon AT3G53590	NI_22F	GAACCAAACAGAACACCTATGTGGA	NI_22R	AAAACGTGTTTGTAAGCAACTCAGC
23	intron AT1G05320	NI_23F	GAAAAATACCGGAAAAGAAACAGATT	NI_23R	CAATATGGAAAACTAACGGCGATT
24	3'UTR AT1G01530	NI_24F	CTCATATTCCACAAGAAAATGATATAAAGC	NI_24R	CCAACACTGCCGTTTGGAT
25	Upstream AT4G34600	NI_25F	TCGATGTATATTCATGCCGATTAAA	NI_25R	TGAAGGAATAAAGCATACGTCGAT
26	intron AT2G32520	NI_26F	CTGAAGCAGAAACGCCATTG	NI_26R	TCTCGCTTATCAGTGCGATCA
27	5'UTR AT4G16830	NI_27F	GGTAGGGGACACTGGACAGG	NI_27R	CCTCAGCATCATCATCCAACA
28	Upstream AT1G61300	NI_28F	GCCATGGACTCTGCTTTTAGC	NI_28R	TCTGGCTTGGCTTCTGTTTG
29	exon AT1G13510	NI_29F	GCTCGGTACGAAGCTCTGTG	NI_29R	TTCAGGGTATGACTTTGAATCTTGA
30	intron AT5G15380	NI_30F	TGAATTTGTGATCTTTGCCATCTT	NI_30R	ACAGAAAGGCTGAATCCCAATC
31	exon AT2G42000	NI_31F	CCCAGCAGATGCTTCTCTCA	NI_31R	CAGGATGCTTTGTCACGACTG
32	exon AT4G31710	NI_32F	TGATTTCTCCCAATGCAACG	NI_32R	GCATCGTAAGCCCTCAATCC

Sanger-sequencing *Onsen* internal primers	
ONSENQ1	ACTACCTTCATTCTCCGGTTC
ONSENQ2	CCTCTTGATGAGTTTTCACCTC
ONSENQ3	TTTCGAATGTTGAGGACAAAC
ONSENQ4	CCCACAACTCTTTTGGTAGTCT
ONSENQ5	TCCCATGTGTCATTCTTTTGTA
ONSENQ6	CTTGTTTTACTTCAATTCCGAGA
ONSENQ7	CTTAACCAAATAGCATGGCATA
ONSENQ8	ACTTGATTTTGCTACTCCAAGC
ONSENQ9	GCAATAGCTTTGGCGAAGAA
ONSENQ10	GCTAGATGATGTGAGAATCATGGA
ONSENQ11	GGAGCTTGATGAATCGGTGA
ONSENQ12	AAGCCCATGTTGAGAAGGAGA
ONSENQ13	CAACTCCAAAGGCTACAAGCTC
ONSENQ14	GCCCCAAGAGCTTGGAATAC

For Supplementary Fig. 3b, initial partial cloning of *Onsen* new insertions in independent *nrpd1-3* plants from transposon display

Primer Forward	Sequence (5'-3')
ONS3643F	TGACGGACATTGGATTGATG

Supplementary Table 3

Sequence polymorphisms in the form of 70-bp sequence ‘addresses’ used for assigning NGS reads to specific genes or each *Onsen* member. The assignment of NGS reads was made by perfect string matching.

> AT2G28390/SAND_adress1
AAAGTCAATTAGAGTCAATGAATTTATCTCAACCTAGCGAAGTCTCTGATGGTAGCCACACCGAATTTA
> AT1G13320/PDF2_adress1
CTATCATTGCTCGTGCTCTTGGAGAGGAGAGGACAAGAAAAGAGTTGATTCCATTTCTTAGTGAGAACA
> AT1G74310/HSP101_adress1
TAGCTGGTGCTTTGATCTCTGATCCCACCGGTATATTTCCTCAAGCAATCTCTAGTGCCGGTGGCGAGA
> AT3G12580/HSP70_adress1
ACGATCAAGGCAACCGCACCACTCCTTCCTACGTTGCTTTCACTGACAGCGAGCGTCTCATCGGGGATG
> AT3G09640/APX2_adress1
GCTAGCCCATGATGCCAACAATGGTCTTGATATTGCCGTTAGGCTTCTTGACCCTATCAAGGAGCTGTT
>AT1G11265/red_adress1
GTTATTAATGGCTAGCTACAAGAAAGATGAACAAAAAGAGAATCATAAGTGGTACCTCGATAGTGGTGCA
>AT1G21945/white_adress1
AATTGTGGGAAGTTTGGACATTAGGCTTCTGAATGTAAAGCTCCTAGCAACAAAAAATTTGAGGAGAAGG
>AT1G48710/violet_adress1
GCTTTCCAAAGGAGTCAAGTTCAAGAGCACAAAAATCGTTGGAGCTAATACATACCGATGTGTGTGGTCC
>AT1G58140/green_adress1
TCGAAGATGGAGGTAAAAGGTAAAGGAAACATTCTTATTCGATTGAAGAATGGAGATCATCAATTTATTT
>AT3G32415/orange_adress1
GAAGACATGTTATTAATGGCTAGTTACAAGAAAGATGAACAAGAAAAGAATCATAAGTGGTACCTCGATA
>AT3G59720/blue_adress1
CTACAATTGCGGGAAGTTTGGACATTATGCTTCTGAATGTAAAGCTCCTAGCAACAAAAAATTTAAGGAG
>AT3G61330/yellow_adress1
GTTATTAATGGCTAGCTACAAGAAAGATGAACAAAAAGAGAATCATAAGTGGTACCTCGATAGTGGTGCA
>AT5G13205/grey_adress1
GTGCAAGTAATCACATGTGCGGGAGAAAAAGTACGTTCGCGGAGCTTGATGAATCGGTGAGAGGAAATGT
>AT1G11265/red_adress2
AATTGGAGTATGTGAAGACACATGATCAAGTAGCCGATTTTTTTACCAAGCCTCTCAAGCGTGAAAACTT
>AT1G21945/white_adress2
ATTGACACACGCTATCACTACATTAGAGAGTGTGTTAGCAAAAAGGACGTGCAATTGGAGTATGTGAAGA
>AT1G48710/violet_adress2
ACAAGAGGAACCAACGAAGATCTTTGTGGATAACAAGTCGGCAATAGCTTTGGCGAAGAACCCGGTCTTC
>AT1G58140/green_adress2
TACATAGTCAAAGGTGAAGAAGACAAAGTCTTGAGACTAAAAAAGGCGCTTTATGGATTAAAACAAGCCC
>AT3G32415/orange_adress2
ACGCTATCACTACATTAGAGAGTGTGTTAGCAAGAAGAACGTGCAATTGGAGTATGTGAAGACACATGAT
>AT3G59720/blue_adress2
TTAGAGAGTGTGTTAGCAAGAAGGACGTGCAATTAGAGTATGTGAAGACACATGATCAAGTAGCCGATAT
>AT3G61330/yellow_adress2
AATTGGAGTATGTGAAGACACATGATCAAGTAGCCGATTTTTTTACCAAGCCTCTCAAGCGTGAAAACTT
>AT5G13205/grey_adress2
ATCAAGTAGCCGATATTTTTACCAAGCCTCTCAAGGGTGAAAACTTTATCAAGATGAGGAGTTTGCTTGG

Supplementary Table 3

>AT1G11265/red_adress3
AAGCAAGATTGGTTGCAAAAGGTTATAGTCAAAGAGTCGGAATTGACTATGACGAGGTATTTGCTCCCGT
>AT1G21945/white_adress3
GTGGAAGATACATCAAATGGATTTCAAGTTGGCCTTCTTAAATGGAGATTTTGAAGAAGAAGTTTACATT
>AT1G48710/violet_adress3
AAGATACAAAGCAAGATTGGTTGCAAAAGGTTATATTCAAAGAGCCGGAATTGACTATGACGAGGTATTT
>AT1G58140/green_adress3
ATAGTCAAAGGTGAAGAAGACAAAGTCTTGAGACTAAAAAAGGCGCTTTATGGATTAAAACAAGCCCCAA
>AT3G32415/orange_adress3
GAAACGGTTAGACTAATCATTTCACTAGCGGCTCAAAACAAGTGGACGATACATCAAATGGATGTCAAGT
>AT3G59720/blue_adress3
GGTTATAGTCAAAGAGCCGGAATTGACTATGACGAGATATTTGCTCCCGTTGCTCGTCTAGAAACGGTTA
>AT3G61330/yellow_adress3
AAGCAAGATTGGTTGCAAAAGGTTATAGTCAAAGAGTCGGAATTGACTATGACGAGGTATTTGCTCCCGT
>AT5G13205/grey_adress3
TGCAAAAGGTTATAGTCAAAGAGCCGGAATTGACTATCACGAGGTATTTGCTCCCGTTGCTCGTCTAGAA

Since AT1G11265/’red’ and AT3G61330/’yellow’ have high sequence similarity, their reads were first pooled together using the same three addresses for both, but subsequently divided to each member using one extra specific address which allowed us to distinguish the count between them. This address was:

>AT1G11265/red_discriminative
AACTACGTTGAAGAAAAAATTCAAGAAGAAGACATGTTATTAATGGCTAGCTACAAGAAAGATGAACAAA
